# Co-expression Mechanism Analysis of Different Tachyplesin I–Resistant Strains in *Pseudomonas aeruginosa* Based on Transcriptome Sequencing

**DOI:** 10.3389/fmicb.2022.871290

**Published:** 2022-04-07

**Authors:** Jun Hong, Xinyang Li, Mengyao Jiang, Ruofei Hong

**Affiliations:** ^1^College of Life Science and Engineering, Henan University of Urban Construction, Pingdingshan, China; ^2^School of International Education, Henan University of Technology, Zhengzhou, China

**Keywords:** co-expressed genes, *P. aeruginosa*, RNA-Seq, SNP, sRNA, tachyplesin I

## Abstract

Tachyplesin I is a cationic antimicrobial peptide with 17 amino acids. The long-term continuous exposure to increased concentrations of tachyplesin I induced resistance in *Pseudomonas aeruginosa*. The global gene expression profiling of tachyplesin I–resistant *P. aeruginosa* strains PA-60 and PA-99 and the sensitive strain *P. aeruginosa* CGMCC1.2620 (PA1.2620) were conducted by transcriptome sequencing to analyze the common underlying mechanism of resistance to tachyplesin I in low- or high-resistance mutants. The co-expression patterns, gene ontology (GO) and Kyoto Encyclopedia of Genes and Genomes (KEGG) pathway enrichment, sRNA target genes, and single-nucleotide polymorphism (SNP) change were analyzed for the co-expressed genes in this study. A total of 661 differentially co-expressed genes under treatments of PA1.2620 vs. PA-99 and PA1.2620 vs. PA-60 (**HL**) were divided into 12 kinds of expression patterns. GO and KEGG pathway enrichment analyses indicated that the enrichment of co-expressed genes was mainly associated with oxidoreductase activity, mismatched DNA binding, mismatch repair, RNA degradation of GO terms, aminoacyl-tRNA biosynthesis, and aminobenzoate degradation pathways, and so forth. The co-expressed resistance-related genes were mainly involved in antibiotic efflux and antibiotic inactivation. Seven co-expressed genes had SNP changes. Some co-expressed sRNAs were involved in *P. aeruginosa* resistance to tachyplesin I by regulating target genes and pathways related to resistance. The common resistance mechanism of *P. aeruginosa* among different mutants to tachyplesin I was mainly associated with the expression alteration of several genes and sRNA-regulated target genes related to resistance; few genes had base mutations. The findings of this study might provide guidance for understanding the resistance mechanism of *P. aeruginosa* to tachyplesin I.

## Introduction

*Pseudomonas aeruginosa* (*P. aeruginosa*) is an important opportunistic pathogen of concern in the healthcare system due to increased rates of resistance to antibiotics or antimicrobial peptides (AMPs). Multidrug resistance in *P. aeruginosa* is increasingly becoming a threat to human health. The bacteria easily form biofilms, which significantly increases bacterial resistance to antibiotics and innate host defense. A number of AMPs have been reported as potential anti-biofilm agents against multidrug-resistant bacteria (Chung and Khanum, 2017; Beaudoin et al., 2018).

During the co-evolution of hosts and bacterial pathogens, bacteria have developed an ability to sense and initiate an adaptive response to AMPs and thus resist their bactericidal activity. *P. aeruginosa* also could develop resistance to a number of AMPs, such as polymyxin (Han et al., 2018). A growing number of *P. aeruginosa* strains demonstrated resistance to AMPs due to mutations in two-component regulatory systems (e.g., PhoPQ, PmrAB, ParRS, and CprRS) (Barrow and Kwon, 2009; Fernández et al., 2012). Changes in drug efflux pumps are one of the main resistance mechanisms of antibiotics or AMPs of *P. aeruginosa*. Some bacteria can use efflux pumps or increase the expression of efflux pumps to mediate resistance against cationic antimicrobial peptides (CAMPs) (Andersson et al., 2016; Puja et al., 2020).

With the rapid development of high-throughput sequencing technology, the study of bacterial transcriptomics can help explore the genes differentially expressed before and after bacterial drug resistance and screen out the non-coding RNAs with regulatory effects. Small RNAs (sRNAs) are important components of post-transcriptional regulation. sRNAs in bacteria have evolved with diverse mechanisms to balance their target gene expression in response to changes in the environment (Dutta and Srivastava, 2018), such as bacterial stress responses, iron uptake, quorum sensing, virulence, and biofilm formation (Wagner and Romby, 2015). sRNAs, in most studied cases, can directly base pair with target mRNA to remodel its expression. As an example, two sRNAs (Sr0161 and ErsA) in *P. aeruginosa* interact with the mRNA encoding the major porin OprD responsible for the uptake of carbapenem antibiotics. The two sRNAs base pair with the 5′ untranslated region (5′UTR) of oprD, leading to an increase in the bacterial resistance to meropenem. Another sRNA Sr0161 positively regulated PagL expression in the absence of Hfq, reducing its pro-inflammatory properties and leading to polymyxin resistance (Zhang et al., 2017). Revealing the post-transcriptional regulation of bacterial drug-resistant genes to screen the existing sRNA in bacteria and study the function of sRNA through transcriptome research is of great significance.

Single-nucleotide polymorphism (SNP) refers to DNA sequence polymorphism caused by nucleotide variations in the genome, including single-base conversion, transposition, and single-base insertion and deletion. In genomic DNA, any base may be mutated. Therefore, SNPs may exist in any sequence of a gene, either in a non-coding sequence or in a gene sequence.

Tachyplesin I, a CAMP with a typical cyclic antiparallel β-sheet structure, was originally isolated from hemocytes of marine horseshoe crabs in 1988 (Nakamura et al., 1988). With potent and broad-spectrum activities against both Gram-positive and Gram-negative bacteria, tachyplesin I has been found as a promising candidate for the development of anti-infection, anti-tumor, and anti-virus drugs (Xie et al., 2016; Kuzmin et al., 2018). Previous studies showed that tachyplesin I could kill bacteria by permeabilizing or damaging the bacterial membrane and acting on intracellular targets in bacteria such as inhibition of DNA, RNA, and protein synthesis and enzyme inactivation (Hong et al., 2013; Kushibiki et al., 2014; Hong et al., 2015; Amiss et al., 2021), and could play a synergistic role. The cytotoxicity of tachyplesin I to mammalian cells was weak in a bacteriostatic dose, and PEGylated tachyplesin I could greatly reduce the cytotoxicity (Imura et al., 2007). Liu et al. (2018) indicated that tachyplesin might kill bacteria by targeting FabG, the conserved β-ketoacyl-acyl carrier protein reductase in unsaturated fatty acid biosynthesis, and provided one possible mechanism to explain how tachyplesin kills bacteria and causes cytotoxicity by targeting membranes.

Bacterial resistance to tachyplesin I has been induced under laboratory conditions, revealing the potential involvement of extracellular protease in mediating tachyplesin I-resistance in Gram-negative bacteria (Hong et al., 2016, 2018). Our results indicated that *P. aeruginosa* resistance to tachyplesin I was mainly related to the reduced entry of tachyplesin I into the bacterial cell and decreased outer membrane permeability (Hong et al., 2020). However, tachyplesin I–induced *P. aeruginosa* mutants were obtained from different generations. The common or specific resistance mechanism of *P. aeruginosa* among different mutants to tachyplesin I has remained elusive.

Therefore, we employed transcriptome sequencing to investigate the differentially co-expressed genes in genome-wide gene expression among tachyplesin I–induced PA-60 and PA-99 mutants and the original strain PA1.2620. Then, the co-expression patterns, gene ontology (GO) and Kyoto Encyclopedia of Genes and Genomes (KEGG) pathway enrichment analysis, annotation and analysis of Comprehensive Antibiotic Resistance Database (CARD), sRNA target genes, and SNP change in the co-expressed genes were analyzed in this study. The findings might enhance our understanding on the common resistance mechanism of *P. aeruginosa* to tachyplesin I.

## Materials and Methods

### Strains, Media, and Growth Conditions

PA1.2620 was provided by China General Microbiological Culture Collection Center (CGMCC; Beijing, China). The minimal inhibitory concentration (MIC) value of tachyplesin I was 10 μg/mL for PA1.2620. Tachyplesin I–resistant mutants (PA-99 and PA-60) were produced in our laboratory by exposure to increasing tachyplesin I concentrations as described previously (Hong et al., 2016, 2020). Namely, PA-60 and PA-99 (naming based on transfer generation) induction strains were developed after 60 and 99 serial transfers in PA1.2620 under long-term selection by exposure to increasing concentrations of tachyplesin. The MIC value of tachyplesin I was 30 and 150 μg/mL for PA-60 and PA-99 mutants, respectively; the two strains had different levels of resistance to tachyplesin I. These strains were cultured in Mueller–Hinton broth (MHB) medium or nutrient agar plates at 30°C.

### Tachyplesin I

Tachyplesin I (>95% purity) was synthesized by Gil Biochemical Co., Ltd. (Shanghai, China), and its sequence was as follows: NH_2_-K-W-C-F-R-V-C-Y-R-G-I-C-Y-R-R-C-R-CONH_2_, including two disulfide bonds (C3–C16 and C7–C12) (10). It was dissolved in sterile water to yield 10 mg/mL stock solution, which was filter-sterilized before use. The peptide solution was freshly prepared on the day of the assay or stored at –20°C for a short period.

### RNA Isolation, cDNA Library Construction, and Transcriptome Sequencing

PA1.2620 original strain and PA-60 and PA-99 mutants were grown overnight for 12 h in 3 mL of MHB medium at 30°C and shaken at 180 rpm. Then, the cultures were refreshed with medium to OD_600 nm_ = 0.2 and grown to the mid-exponential phase (OD_600 nm_ = 1.0) until the time of harvesting. The extraction and purification of the total RNA from the cells were carried out using a bacterial total RNA purification kit (Sangon Biotech Co., Ltd., Shanghai, China). The quality, quantity, and integrity of extraction were analyzed using a NanoDrop 2000 spectrophotometer (Thermo Fisher Scientific, MA, United States), gel electrophoresis, and an Agilent 2100 Bioanalyzer (Agilent Technologies Inc., CA, United States), respectively, to ensure the use of total RNA sequencing for transcriptome analysis. All experiments were performed in triplicate.

After the samples of total RNA passed the test, the library construction was carried out. The main procedures for library construction were the same as in the previous study (Hong et al., 2020). The RNA-Seq libraries of nine samples were prepared and subsequently sequenced using an Illumina HiSeq 2500 Platform (Illumina Inc., CA, United States) following the manufacturer’s protocols. The construction of the libraries and the transcriptome sequencing were performed by the Biomarker Biotechnology Corporation (Beijing, China).

### Analysis and Assembly of RNA-Seq Data

Generally, the raw reads contained tiny minority primer sequences, adapter sequences, sequencing connectors, and other potential contaminants. Prior to subsequent analysis, the clean reads were filtered from the raw reads by removing the reads with only adaptor and unknown nucleotides.

The clean reads obtained from RNA-Seq were mapped on the reference genome of *P. aeruginosa* PAO1 using Bowtie-2 software, and only mapped data were used for subsequent analysis (Langmead et al., 2009). Data analysis and base-calling were undertaken using Illumina sequencing software (Illumina Inc., CA, United States).

### Gene Expression Analysis

Each UniGene cluster was compared via UniGene database using Bowtie-2 software to evaluate the expression level of all reads and normalized into Reads Per Kilobase of transcript per Million reads mapped (RPKM) values using the following formula: RPKM = (Map the UniGene cluster)/[Map all UniGene clusters (million)] × [The length of UniGene cluster (kb)]. Also, differentially expressed genes (DEGs) with a false discovery rate (FDR < 0.01) and a fold change of ≥ 2 were selected for analysis according to the DESeq software (Wang et al., 2010).

### Functional Annotation and Enrichment Analysis of Differentially Expressed Genes

The functional annotation of each UniGene cluster was searched against various protein databases and identified using annotation information of the given UniGene cluster that had the highest sequence similarity with the tested one. The Blast2GO program and Web Gene Ontology Annotation Plot (WEGO) software were also employed to obtain GO annotations and the distribution of gene functions for each UniGene cluster using a value of less than 10^–5^ (Ashburner et al., 2000). The assignment of each UniGene cluster to different pathways was carried out by searching in the KEGG database using KEGG Automatic Annotation Server (Kanehisa et al., 2004). DEGs were annotated in the CARD using Resistance Gene Identifier (RGI) to find out the antibiotic resistance genes (Jia et al., 2017).

### Analysis of Differentially Expressed Small RNA and Regulatory Target Genes

The reads mapped on the reference genome sequence were checked to determine whether that region could form a hairpin structure and was located on the arm. The prediction result was obtained after screening using the miRDeep2 software. The differentially expressed sRNAs in different treatment groups were screened out. The differential sRNA target gene data were processed to identify the DEGs, and GO function and KEGG pathway enrichment analyses were performed.

### Single-Nucleotide Polymorphism Analysis of Differentially Expressed Genes and Small RNA Target Genes

Based on the results of reads mapped on reference genome sequences of each sample using the Bowtie software, the Genome Analysis ToolKit (GATK) software was used to identify a single-base mismatch between the sequenced sample and reference genome and search for potential SNPs in gene regions. Comparison of the sample sequence with the reference transcriptome sequence and statistical comparison were performed on the SNPs in each sample of the control and treatment groups, and the statistical results of their types and numbers were analyzed. The location information of genes was extracted from the gff files of *P. aeruginosa* using the GFF information filtering tool based on the gene ID of the Biomarker Cloud platform. The number and types of SNPs generated in DEGs were obtained by comparing the location information of SNPs with the corresponding location information of genes. The obtained data were analyzed mainly through the Cloud platform of Biomarker.

## Results

### Sequence Analysis and Assembly

The transcriptome sequencing of nine cDNA libraries was performed using total RNA extracted from PA-99 and PA-60 strains and the original strain PA 1.2620. After filtering the raw data and passing quality control, the clean data of each sample were no less than 2.11 Gb, the GC content of clean data was more than 62.57%, and the Q30 base reached more than 95.42% (Supplementary Table 1). These data were selected as high-quality reads for further analysis.

The evaluation of transcriptome sequencing data of nine samples, including those mapped to the reference genome, randomness testing of mRNA fragmentation, saturation testing of data, and distribution of reads on the reference genome were performed. The results revealed that the transcriptome data obtained were ideal and reliable in terms of quality. The statistical reads of clean data mapped to reference genome mapping statistical data are presented in Supplementary Table 2.

The transcriptome sequencing results revealed a correlation between one biological replicate (PA1.2620^1^) and the other two replicates with low correlations (0.4689, 0.4735) of the original strain PA1.2620, whereas *R*^2^ between the other two samples was 0.9931 (Supplementary Table 3). In addition, among the three biological replicates of PA-60 strain having high correlations, one of the biological replicates (PA-60^1^) was far different from the overall dispersion of other samples. Considering the accuracy of the results, we deleted one biological replicate of PA1.2620 with poor correlation and one biological replicate of PA-60 that was different from the other two samples (Supplementary Table 3 and Supplementary Figure 1).

### Gene Expression Analysis

**FIGURE 1:**
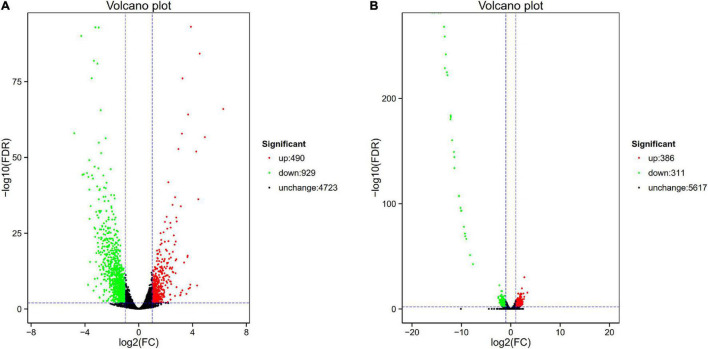
Volcano plot of differential expression in PA1.2620 different strain. **(A)** PA1.2620 original strain vs. PA–60 mutant. **(B)** PA-60 strain vs. PA–99 mutant. Each point in volcano plot represents one gene, and the abscissa represents the logarithm of the expression difference fold of one gene. The vertical axis represents the negative logarithm of the error detection rate. The green dots represent down-regulated genes, the red dots represent up-regulated genes and the black dots represent unchanged genes.

**FIGURE 2:**
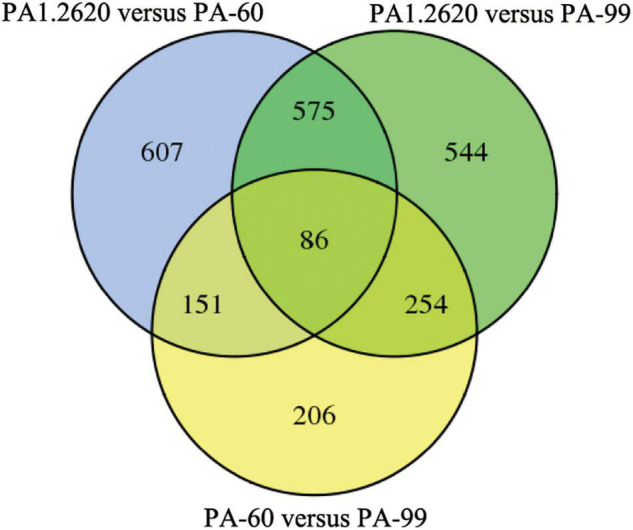
Venn diagram of DGEs in different treatments. Venn diagram represents the number of DGEs and overlap between comparison groups. The sum of the numbers in each large circle represents the total number of differential genes in the comparison. The overlapped part of the circle represents the common differential genes between the combinations.

We selected tachyplesin I–induced 99 transfer strain (PA-99 mutant), 60 transfer strain (PA-60 mutant), and the original strain PA1.2620 to perform expression profiling analysis so as to investigate the changes in transcription levels before and after PA1.2620 resistance to tachyplesin I (Figures 1, 2).

As illustrated in Figure 1A, the PA-60 mutant had 1419 DEGs, including 490 (34.53%) upregulated genes and 929 (65.47%) downregulated genes, compared with the original strain PA1.2620; the log2FC values of expression were mainly distributed between –2.5 and 2.5. Besides, the expression of 37 genes was downregulated with more than sixfold changes. The top three downregulated genes were *Novel_815*, *Novel_838*, and *Novel_43*. According to Basic Local Alignment Search Tool (BLAST) analysis, the homologous protein of *Novel_815* gene (log2FC = –4.793) was ferredoxin. The *Novel_838* (log2FC = –4.275) was permease protein MsbA. The *Novel_43* gene had no homologous proteins. The most significantly upregulated gene was *fruA*, and its homologous protein was the phosphotransferase system (PTS) fructose-specific IIBC component. Whether PA-60 low resistance to tachyplesin I was associated with the changes in highly expressed genes needs to be further explored.

The PA-99 mutant had 1459 DEGs, including 672 (46.06%) upregulated genes and 787 (53.94%) downregulated genes, compared with the original strain PA1.2620; the log2FC values of expression levels were mainly distributed between –5 and 5.

The study further analyzed the difference in gene expression between different mutants. The PA-99 mutant had 697 DEGs, including 386 upregulated genes and 311 downregulated genes, compared with the induced strain PA-60 (Figure 1B). A total of 29 genes had downregulated expression with more than tenfold changes. The top five genes were *Novel_392*, *PA2754a*, *PA2765*, *PA2764*, and *PA2758*, all with downregulated expression. According to BLAST analysis, the Novel_392 and PA2754a had no homologous proteins. The homologous protein of PA2765 was peroxidase. The homologous proteins of PA2764 and PA2758 belonged to the alpha/beta hydrolase superfamily and LysR family of transcriptional regulators.

Common or specific DEGs in different strains were shown in the Venn diagram (Figure 2). A total of 661 co-expressed genes DEGs were found in the treatments of PA1.2620 vs. PA-60 and PA1.2620 vs. PA-99 (**HL**). A total of 237 co-expressed genes were under the treatments of PA1.2620 vs. PA-60 and PA-60 vs. PA-99, while 340 co-expressed genes were under treatments of PA1.2620 vs. PA-99 and PA-60 vs. PA-99. Moreover, 86 common DEGs were found among the 3 treatment groups. The results showed DEGs and co-expressed genes in different strains of induced transfers. However, 785 specific DEGs were identified in the PA1.2620 vs. PA-60 treatment group and 798 specific DEGs in the PA1.2620 vs. PA-99 treatment group. The result indicated that different tachyplesin-induced transfers influenced the gene expression levels of DEGs of *P. aeruginosa* mutants compared with the original strain. In other words, the expression levels of DEGs changed to varying degrees with the increase in *P. aeruginosa* resistance to tachyplesin I. We further analyzed the role of the co-expressed genes in the resistant mechanism of *P*. aeruginosa to tachyplesin I.

### Analysis of Co-expression Patterns of HL Treatments

**FIGURE 3:**
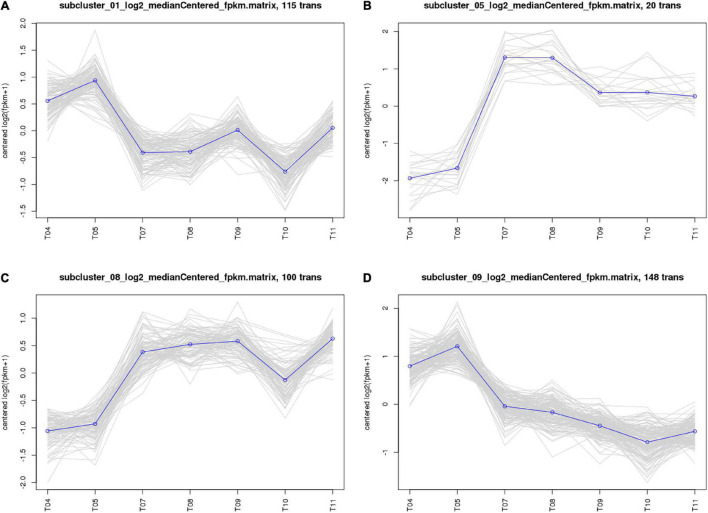
Analysis of co-expressed patterns of HL treatments. **(A)** Subcluster_01. **(B)** Subcluster_05. **(C)** Subcluster_08. **(D)** Subcluster_09. The abscissa represents different sample, the vertical axis represents centered log2 (fpkm + 1). Four clusters were identified based on expression levels in seven different samples. T04–T05 are the two biological duplicates of the original strain PA1.2620; T07–T08 are the two biological duplicates of the PA-60 strain; T09–T11 are the three biological duplicates of PA-99 strain.

The 661 common DEGs in treatments of HL were analyzed to explore the co-expression patterns among PA1.2620 different mutants. Using the K-means clustering algorithm in BMKCloud Platform, 661 co-expressed genes were grouped into 12 clusters (subcluster_01–12) with the increasing resistance to tachyplesin or the increasing tachyplesin-induced transfers. The gene expression levels of six subclusters mainly had an increasing trend, while others had decreased trends. The part results (subcluster 01, 05, 08, and 09) are shown in Figure 3. These gene expression levels of subcluster 06 (75 genes), subcluster 07 (7 genes), subcluster 09 (148 genes), and subcluster 12 (44 genes) primarily followed similar patterns of expression with the increasing resistance to tachyplesin, which decreased from T04 (PA1.2620) to T09 and reached a steady state in T09–T11 (PA-99). These gene expression levels of subcluster 01 (115 genes) and subcluster 11 (63 genes) had primarily similar patterns with the increasing resistance to tachyplesin, which obviously decreased from T04 to T07–T08, and slightly increased from T08 to T09, then decreased in T10. In contrast, the gene expression levels of subcluster 03 with 7 genes and subcluster 05 with 20 genes showed similar patterns, which both obviously increased from T04 to T07–T08 (PA-60) and decreased in T09. Then reached a steady state in T10–T11. The gene expression levels of subcluster 08 with 100 genes increased from T04 to T05 to T07, reached a steady state in T07–T09, and then slightly fluctuated in T10–T11. The co-expression patterns indicated that different tachyplesin-induced transfers influenced the gene expression level in *P. aeruginosa.*

### Functional Classification and Enrichment Analysis of the Co-expressed Genes of HL

**FIGURE 4:**
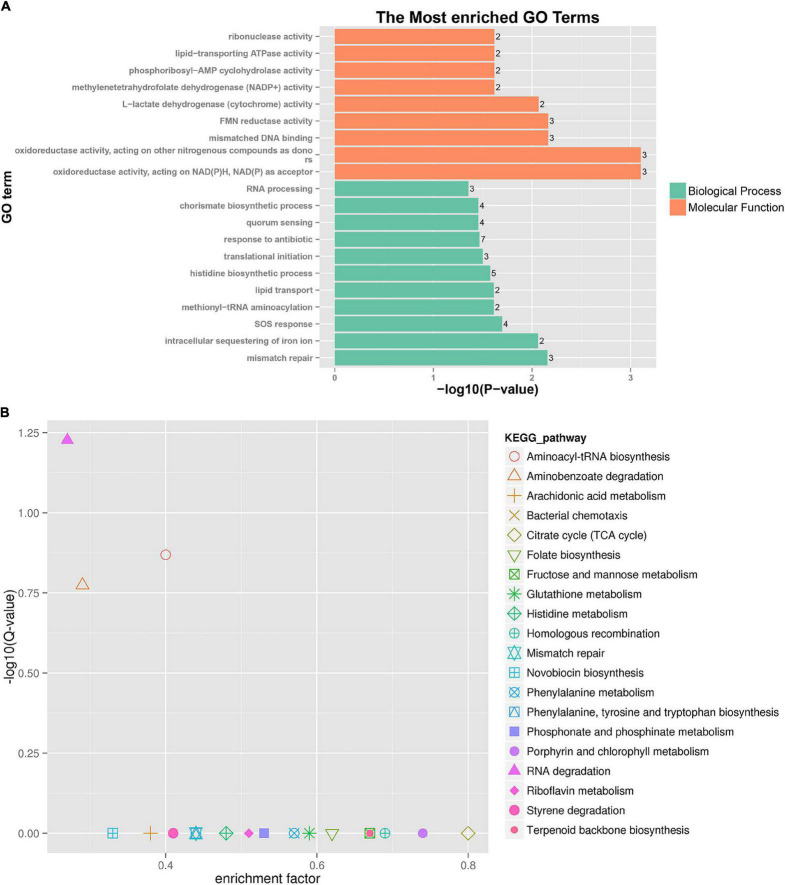
GO and KEGG pathway enrichment analysis of DEGs between HL treatments. **(A)** GO enrichment. The bigger the value of –log10 (*p*-value), the more enriched. *p*-value < 0.05 is the significant difference, and *p*-value < 0.01 is the most significant difference. The ordinate is “GO term.” **(B)** KEGG pathway enrichment. Enrichment factor = Amount of all genes/Amount of DEGs enriched in the pathway in the background gene set. The smaller the enrichment factor, the more significant. The same below.

A further functional classification and enrichment analysis of 661 common DEGs between **HL** was performed using GO and KEGG databases. The GO annotation classification indicated that common DEGs were enriched in cellular components, molecular functions, and biological processes; it was subdivided into 46 GO_classify2 functional terms. As shown in Figure 4A, the top 20 GO terms were significantly enriched (*P*-value < 0.05) in the biological process and molecular function. Detailed information about the enriched GO terms was provided in Supplementary Table 4. Of these enriched GO terms, nine terms were related to molecular function. The top four enriched GO terms included “oxidoreductase activity, acting on NAD(P)H, NAD(P) as acceptor,” “oxidoreductase activity, acting on other nitrogenous compounds as donors,” “mismatched DNA binding,” and “FMN reductase activity.” Further, 11 terms related to biological process mainly included “mismatch repair,” “intracellular sequestering of iron ion,” “SOS response,” “quorum sensing,” and so forth. The GO enrichment indicated that tachyplesin significantly affected oxidoreductase activity and mismatched DNA binding in *P. aeruginosa*.

A total of 186 DEGs were assigned to 71 KEGG pathways. The KEGG pathway analysis revealed 20 over-represented pathways (*P*-value < 0.5), as shown in Figure 4B and Supplementary Table 5. The top three enriched pathways (corrected_*P*-value < 0.5) were RNA degradation (ko03018), aminoacyl-tRNA biosynthesis (ko00970), and aminobenzoate degradation (ko00627). Eight DEGs were involved in RNA degradation pathways, 12 DEGs in aminoacyl-tRNA biosynthesis pathways, and 7 DEGs in aminobenzoate degradation pathways. In addition, the two-component system (ko02020, 18 DEGs), lipopolysaccharide biosynthesis (ko00540, 2 DEGs), and amino sugar and nucleotide sugar metabolism (ko00520, 3 DEGs) pathways were involved in several known CAMP resistance DEGs. The beta-lactam resistance (ko01501, 3 DEGs), flagellar assembly (ko02040, 2 DEGs), bacterial secretion system (ko03070, 8 DEGs), and bacterial chemotaxis (ko02030, 10 DEGs) were also related to resistance. The enriched pathway showed that *P. aeruginosa* resistance to tachyplesin I resulted in some alterations in several pathways related to resistance.

### Annotation and Analysis of Co-expressed Resistance Genes of HL

**FIGURE 5:**
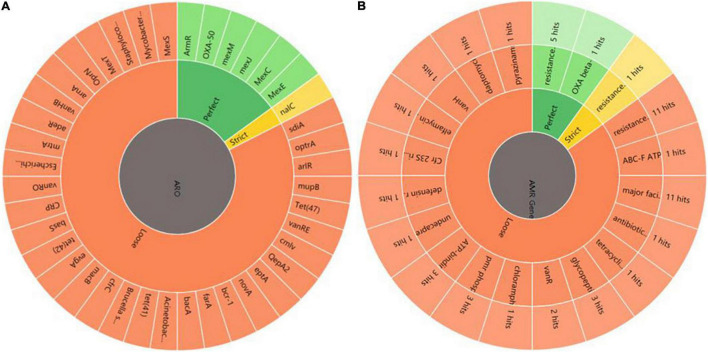
Annotation and analysis of co-expressed resistant genes between HL. **(A)** AMR genes. ARO represents antibiotic resistance ontology. The outer ring represents antibiotic resistance genes in the figure. **(B)** AMR gene family. The inner ring represents the anti-microbial resistance (AMR) gene families and the outer ring represents hits the numbers of resistance genes predicted.

First, DEGs were annotated in CARD using RGI to select the known antibiotic resistance genes. Under RGI criteria with the perfect, strict, complete genes only which is the most commonly used, seven co-expressed genes in **HL** treatments were mainly involved in antibiotic efflux for resistance–nodulation–cell division (RND) antibiotic efflux pump (six genes, including *armR*, *nalC*, *PA1435*, *PA3677*, *mexC*, and *mexE*) and antibiotic inactivation (PA5514 coding for OXA betalactamase), as shown Figure 5A. The result showed that *P. aeruginosa* resistance to tachyplesin I mainly involved the overexpression of the RND efflux pump, which was independent of the induction time.

Under RGI criteria with the perfect, strict and loose, and complete genes only which can be used for reference to predict drug resistance genes, 74 co-expressed potential resistance genes had high homology with 46 known antibiotic resistance genes in treating **HL** (Figure 5A). The common resistance mechanisms of tachyplesin in *P. aeruginosa* mainly involved antibiotic efflux pump (44 genes), antibiotic inactivation (11 genes), antibiotic target alteration (17 genes), and antibiotic target protection (2 genes). The mechanism of the antibiotic efflux pump involved these genes of RND, major facilitator superfamily (MFS), ATP-binding cassette (ABC), and multidrug and toxic compound extrusion (MATE) transporter families. Their expression levels were most upregulated with log2FC ≤ 2 fold changes in the RND efflux pump genes. The mechanism of antibiotic target alteration was mainly involved in 11 anti-microbial resistance (AMR) gene families. The mechanism of antibiotic inactivation was mainly involved in beta-lactamase, fosfomycin thiol transferase, and chloramphenicol phosphotransferase of AMR gene families.

The top three members of AMR gene families were RND, MFS, and ABC antibiotic efflux pump, detected using homologous alignment in RGI analysis. AMR gene families mainly included 23 potential genes with high homology to 17 members of the RND efflux pump family, 16 potential genes with high homology to the 11 members of the MFS efflux pump family, 4 potential genes (*PA4223*, *Novel_141*, *rbsA*, and *PA3376*) with high homology to the 3 members of the ABC efflux pump family, and 3 potential genes (*fmt*, *PA2480*, and *PA4517*) with high homology to the 3 members of the pmr phosphoethanolamine transferase family. Three genes (gene ID: *Novel_737, Novel_736, and Novel_612*) were involved in the antibiotic resistance isoleucyl-tRNA synthetase (ileS) family (Figure 5B). The MFS efflux pump family was involved in more DEGs in this study. However, the function of MFS genes in *P. aeruginosa* resistance to tachyplesin was unclear and needed further investigation.

### Single-Nucleotide Polymorphism Analysis

The data statistical analysis of SNP loci numbers, proportion and types of transition, and transversion and heterozygosity of SNPs on the samples showed that the frequency of SNPs was relatively consistent in each sample relative to the control PA1.2620 genome, in which the frequency of transition types was much higher than that of transversion types. Among the transition types, the most conversions were from A to G, followed by C to T. With the increasing tachyplesin resistance from PA-60 to PA-99 strains, the transition frequency tended to decrease while the transversion frequency tended to increase (Table 1). Also, differences were found in the frequency of occurrence between different groups of biological duplicates in the same sample.

**TABLE 1:** Statistical table of mutation sites and mutation category of SNP.

SNP category Name	PA-60 sample		PA-99 sample		
	PA-60^2^	PA-60^3^	PA-99^1^	PA-99^2^	PA-99^3^
Transition number	153	363	105	196	91
A/G	81	208	56	107	46
C/T	72	155	49	89	45
Transversion number	31	32	33	29	34
A/C	7	10	7	5	7
A/T	7	5	9	7	6
C/G	11	10	9	11	14
G/T	6	7	8	6	7
T/C, G	1	0	1	1	1
Total SNP number	185	395	139	226	126
Transition%	82.70	91.90	75.54	86.73	72.22
Transversion%	16.76	8.10	23.74	12.83	26.98
Heterozygosity%	0.54	0.00	0.72	0.44	0.79

*PA-60^2^ and PA-60^3^ are two biological duplicates of the PA-60 strain, while PA-99^1^, PA-99^2^, and PA-99^3^ are three biological duplicates of the PA-99 strain.*

### Single-Nucleotide Polymorphism Analysis of Co-expressed Genes Between HL

Using the PA1.2620 strain as the control group, the PA-60 and PA-99 strains were compared to find the common SNP changes of DEGs. For these SNP genes, the base mutations that occurred only in one biological repeat were filtered out. A total of 22 DEGs (12 upregulated and 10 downregulated) had SNP changes between PA1.2620 and PA-99 strains, while 14 DEGs had SNP changes between PA1.2620 and PA-60 strains. Seven co-expressed genes had SNP changes, with four upregulated and three downregulated, as shown in Table 2.

**TABLE 2:** SNP change of the co-expressed genes of **HL**.

Gene #ID or name	Protein_name	PA1.2620 vs. PA-60 (log2FC)	PA1.2620 vs. PA-99 (log2FC)	Regulated	Start	End	SNP number	Snp
*pvdL*	Peptide synthase	1.10009	1.15621	Up	2,707,666	2,720,694	2	2712019C > A; 2715424T > C
*Novel_244*	–	–1.10470	–1.71631	Down	1,720,641	1,732,256	1	1732254T > C
*PA1938*	Hypothetical protein	–1.03418	–1.95428	Down	2,118,926	2,119,747	1	2119153T > C
*Novel_745*	–	1.86917	2.58303	Up	5,130,955	5,135,726	1	5130955T > C
*PA0193*	Hypothetical protein	1.39140	1.186966	Up	221,585	222,487	1	222358C > T
*exoY*	Adenylate cyclase	–1.08212	–1.75024	Down	2,410,344	2,411,480	1	2411150G > T
*vreR*	Sigma factor regulator VreR	2.20828	1.68468	Up	735,487	736,446	1	735564C > T

Among the seven genes with SNP change, two were novel genes. SNPs mainly occurred in log2FC values of gene expression from onefold to threefold change. However, only two base mutations occurred in the *pvdL* gene. According to BLAST analysis, *Novel_745*, *PA1938* and *PA0193* genes were described as hypothetical proteins, while a homology of *Novel_244* gene was 2-oxoglutarate dehydrogenase.

### Analysis of Co-expressed Small RNA of HL

A total of 17 differentially expressed novel sRNAs were found under treatments of PA1.2620 vs. PA-60, and 11 differentially expressed sRNAs under treatments of PA1.2620 vs. PA-99, as detected by RNA sequencing. Six novel sRNAs with downregulated expression were co-expressed under treatments of **HL,** as shown in Table 3. Considering different sRNAs with the same target genes, 91 upregulated differentially expressed target genes were found after filtering out the duplicates.

**TABLE 3:** Co-expressed differentially expressed sRNA of **HL**.

sRNA (ID)	Gene length (nt)	PA1.2620 vs. PA-60 (log2FC)	PA1.2620 vs. PA-99 (log2FC)	Regulated
Novel_33	188	−2.904338527	−3.101050802	Down
Novel_377	378	−1.584854106	−2.065876992	Down
Novel_583	78	−3.066999834	−3.461144761	Down
Novel_584	116	−1.973275535	−2.305895532	Down
Novel_6	94	−1.43082481	−1.576755833	Down
Novel_853	117	−1.028188198	−1.507421364	Down

### Functional Enrichment Analysis of Co-expressed Small RNA Target Genes

**FIGURE 6:**
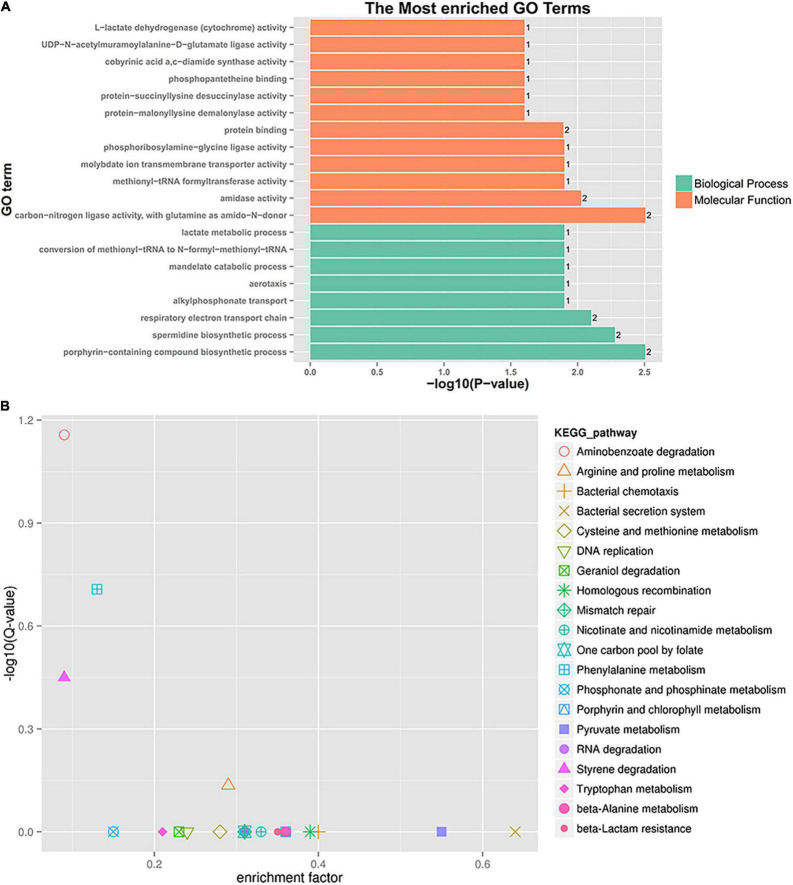
GO enrichment and KEGG pathway analysis of co-expressed sRNA target genes. **(A)** GO enrichment. **(B)** KEGG pathway enrichment.

A further functional classification and enrichment analysis of six co-expressed sRNA target genes (91) for **HL** was performed using GO and KEGG databases. As shown in Figure 6A, the top 20 GO terms were significantly enriched (*P*-value < 0.05) in the biological process and molecular function. Detailed information about the enriched GO terms is provided in Supplementary Table 6A. Of these enriched 20 GO terms, 12 terms were related to the molecular function and 8 terms were related to the biological process. The top four enriched GO terms (*P*-value < 0.01) included “carbon-nitrogen ligase activity, with glutamine as amido-N-donor (two genes),” “porphyrin-containing compound biosynthetic process (two genes),” “spermidine biosynthetic process (two genes),” and “respiratory electron transport chain (two genes).”

A total of 91 differentially co-expressed sRNA target genes were assigned to 29 KEGG pathways. The KEGG pathway analysis revealed the top 20 pathways (*P*-value < 0.5), as shown in Figure 6B and Supplementary Table 6B. The top four enriched pathways included aminobenzoate degradation (ko00627, three target genes), phenylalanine metabolism (ko00360, three target genes), styrene degradation (ko00643, two target genes), and arginine and proline metabolism (ko00330, four target genes). Besides the first 20 pathways, the two-component system (ko02020, three target genes), aminoacyl-tRNA biosynthesis (ko00970, one target gene), ABC transporters (one target gene), and glutathione, nitrogen, and sulfur metabolism were also detected. The enriched pathway showed that some sRNA played a role in *P. aeruginosa* resistance to tachyplesin I by regulating the pathway changes of target genes, such as aminobenzoate degradation, amino acid metabolism, bacterial chemotaxis, two-component system, and so on.

### Single-Nucleotide Polymorphism Change of the Co-expressed Small RNA Target Genes

The SNPs of six co-expressed sRNA of **HL** showed no change. The target genes of 91 differentially co-expressed sRNAs were analyzed for changes in SNP. A total of three target genes (upregulated) showed SNP changes after removing SNPs that occurred only in one biological replicate gene (Table 4). The expression levels of two sRNA target genes (*vreR* and *Novel_745*) were different in PA1.2620 vs. PA-60 and PA1.2620 vs. PA-99. Few SNP changes were found in sRNA target genes, and most of them occurred in target genes with low differential expression. Only the *pvdL* gene had two site mutations, while the other two genes had one site mutation. Also, the *pvdL* encoding peptide synthase gene was mainly involved in the synthesis of *P. aeruginosa* iron carrier and the biological synthesis of lipids; it participated in the molecular function of catalytic activity. How sRNA participates in the function of these target genes remains to be further explored.

**TABLE 4:** SNP change of the co-expressed sRNA target genes of **HL**.

Gene ID or name	PA1.2620 vs. PA-60 (log2FC)	PA1.2620 vs. PA-99 (log2FC)	Regulated	Start	End	SNP number	Snp
*pvdL*	1.100094764	1.156212	UP	2,707,666	2,720,694	2	2712019C > A; 2715424T > C
*vreR*	2.208278525	1.684682	Up	735,487	736,446	1	735564C > T
*Novel_745*	1.86916584	2.583033	Up	5,130,955	5,135,726	1	5130955T > C

## Discussion

By analyzing DEGs in drug-resistant strains, this study might help researchers select from several DEGs closely related to bacterial resistance genes and participating in metabolic pathways, thus providing new ideas for revealing the mechanism of bacterial drug resistance. sRNA is a class of newly discovered gene expression regulators that control cellular physiological functions in response to various environmental changes by pairing with target mRNA or target protein, especially in the development of bacterial drug resistance. These differentially expressed sRNAs mainly act on corresponding target mRNAs and then play their regulatory role. At the genomic level, SNP can be used to analyze the association between complex traits and reveal the genetic mechanism of related traits. Drug resistance caused by gene mutations is a process of long-term accumulation. Different from transcriptome, the genome is relatively stable and not susceptible to mutation. After special induction treatment, directional mutations can be generated to cope with selective pressure in the environment. These mutations may help adapt to the new environment after environmental changes in the strain and may have nothing to do with specific mutations in genes related to drug resistance and virulence. Therefore, based on transcriptome sequencing, the analysis of DEGs, sRNA target genes, and SNP changes among tachyplesin I–resistant strains and the original strain can be used to predict the common cause of *P. aeruginosa* resistance to tachyplesin at the transcriptome and genome levels, to provide certain theoretical support for the generation of *P. aeruginosa* resistance to tachyplesin.

This study attempted to analyze further the common underlying mechanism between different mutants resistant to tachyplesin I using mRNA expression profiling. From the transcriptome data, we found that several common genes exhibited significantly altered expression. To validate the results of transcriptome sequencing, 10 DEGs from co-expressed genes of HL were randomly selected for RT-qPCR. All the selected DEGs showed concordant expression patterns between the RNA-Seq and the results of RT-qPCR. The part results of 10 DEGs from PA1.2620 vs. PA-99 for RT-qPCR had been published in previous study (Hong et al., 2020). A total of 661 differentially co-expressed genes were divided into 12 kinds of expression patterns, which might be beneficial for understanding the resistance mechanism of *P. aeruginosa* to tachyplesin I.

Aminoacyl-tRNA biosynthesis was a significantly enriched pathway in treating **HL** with 12 DEGs (1 upregulated and 11 downregulated). The result showed that *P. aeruginosa* resistance to tachyplesin involved the expression of some genes of aminoacyl-tRNA synthesis pathways, but no gene had SNP. The function of aminoacyl-tRNA synthesis is to precisely match amino acids with tRNAs containing the corresponding anticodon. Besides having essential roles in protein synthesis and Gram-positive peptidoglycan cross-linking or membrane phospholipid modification in some bacteria, aminoacyl-tRNAs are also involved in pathways directly implicated in antibiotic biosynthesis and resistance (Shepherd and Ibba, 2013). For example, MprF proteins mediated drug resistance to control the permeability of the cell wall to cationic antimicrobials by catalyzing the aminoacylation of inner membrane lipids (Ernst and Peschel, 2011). Aminoacylation of membrane lipids adjusts the net negative charge of the membrane bilayer in a manner dependent on the aminoacyl-tRNA substrate.

*P. aeruginosa* has Ala-phospholipid phosphatidyl-glycerols (PG), which can aminoacylate phosphatidylglycerol with Ala and thus neutralize the overall charge of the membrane to enhance the resistance to a multitude of negatively charged β-lactam antibiotics. Ala-PG also has a significant influence on the permeability and fluidity of the lipid bilayer. The *PA0920* gene, as the mprF ortholog, was responsible for alanyl-PG synthesis (Klein et al., 2009), and the tRNA-dependent enzyme was located in the inner membrane. ORF PA0919 coded for an alanyl-PG hydrolase that was anchored to the periplasmic surface of the inner membrane (Arendt et al., 2013). PA0919 was termed alanyl-PG hydrolase. In our study, *PA0919* was upregulated in PA-60 (Log2FC = 2.28) and PA-99 (Log2FC = 3.749); the upstream gene *PA0920* was also upregulated in PA-60 (Log2FC = 2.74) and PA-99 (Log2FC = 4.20). The expression trends of two genes showed that the *PA0920* gene positively regulated *PA0919* gene expression, and the expression level gradually increased with the increased levels of drug resistance in mutant strains. Our previous results indicated that the PA-99 strain resistant to tachyplesin I was mainly related to the reduced entry of tachyplesin I into the bacterial cell due to the overexpression of the efflux pump, in addition to a decrease in outer membrane permeability (Hong et al., 2020). We inferred that different *P. aeruginosa* resistance to tachyplesin might be related to a decrease in the overall net negative charge of the membrane or a decrease in the permeability due to lipid modification.

One of the mechanisms of bacterial resistance to AMPs is the reduced entry of the AMPs into the bacterial cell. The transcriptional regulation of HL indicated that several genes encoded by the outer membrane efflux protein or multidrug resistance protein were all upregulated, three genes (ID *Novel_156*, *Novel_157*, *Novel_443*) encoded by outer membrane porin were downregulated, and only one opdB gene was upregulated. The results showed that the common mechanism of *P. aeruginosa* resistance to tachyplesin was also related to overexpression of genes encoding the outer membrane porins and multidrug resistance protein.

Drug efflux through efflux pumps is one of the main resistance mechanisms of *P. aeruginosa* against antibiotics or AMPs (Puja et al., 2020; Zahedi bialvaei et al., 2021). Some bacteria could increase the expression of efflux pumps to mediate resistance against CAMPs (Andersson et al., 2016). The efflux pumps of the RND family could be directly related to the efflux of antibiotics in *P. aeruginosa*. MFS and ABC families were also two important superfamilies of membrane transporters, playing a significant role in substance exchange, energy metabolism, and drug resistance. In the present study, the co-expressed DEGs of **HL** involved in high-homology resistance genes were found in ABC, MFS, RND, and MATE transporter efflux pump families. Most genes were encoded by RND and MFS efflux pumps, whereas only one gene related to the MATE efflux pump was found, indicating that the RND and MFS antibiotic efflux pumps had an important role in *P. aeruginosa* resistance to tachyplesin, especially RND efflux pumps.

A large number of studies also found that drug efflux pumps could further enhance drug resistance by promoting biofilm formation. The drug resistance mechanism of biofilm formation played a pivotal role in *P. aeruginosa*. The biofilm formation pathway in *P. aeruginosa* mainly involved quorum sensing, bacterial secretion system, and flagellar assembly. We found that several co-expressed genes in treating **HL** and co-expressed sRNA target genes were assigned to flagellar assembly and bacterial secretion system pathway. In a previous study, PA-99 and PA-60 mutants formed biofilms easily compared with the original strain PA1.2620 with increased resistance to tachyplesin; the contents of extracellular polysaccharides were also higher than that in the original strain (data not shown). When the content of exopolysaccharide reached a certain level, it promoted biofilm formation. The results further indicated that the common mechanism of *P. aeruginosa* resistance to tachyplesin I could be involved in expressing of biofilm-related genes. However, whether the resistance mechanism of efflux pumps was related to biofilm formation needs to be further investigated.

A two-component regulatory system plays a substantial role in the pathogenicity, virulence, biofilm formation, and drug resistance in *P. aeruginosa* (Bhagirath et al., 2019). In the present study, we detected 18 co-expressed DEGs for a two-component regulatory system under the treatment of **HL** and 3 co-expressed sRNA target genes, including some regulated resistance genes, such as response regulator phoP (gene ID: *Novel_194*) and alkaline phosphatase encoded by phoA (gene ID: *Novel_498, 499*). The result showed that a two-component regulatory system might also play a significant role in *P. aeruginosa* resistance to tachyplesin, and sRNA might also be involved in regulating the expression of the two-component system. A previous study demonstrated the importance of regulatory genes, such as phoP-phoQ and pmrA-pmrB, as well as the addition of aminoarabinose to lipid A (Barrow and Kwon, 2009).

In summary, mutants resistant to tachyplesin I obtained at different induction times had 661 co-expressed DEGs under the treatment of **HL**, which were divided into 12 kinds of expression patterns. Some co-expressed genes were regulated by sRNA. A few SNPs with relatively low expressed genes had base mutations, which were mainly related to iron metabolism, adenylate cyclase, and encoding of putative proteins. Several known CAMP resistance pathways and antibiotic resistance mechanisms might be involved in the common potential mechanism of *P. aeruginosa* resistance to tachyplesin I. The findings might enhance our understanding of the common resistance mechanism of different *P. aeruginosa* strains to tachyplesin I. The specific resistance mechanism of *P. aeruginosa* among different mutants to tachyplesin I needs further exploration.

## Data Availability Statement

The original contributions presented in the study are included in the article/Supplementary Material, further inquiries can be directed to the corresponding author/s.

## Author Contributions

JH and XL conducted the data analysis and wrote the manuscript. JH conceived the idea, collected the data, and aided with the writing. MJ and RH contributed to refine and reorganized the data used in the manuscript and figure preparation. All authors have read and approved the manuscript.

## Conflict of Interest

The authors declare that the research was conducted in the absence of any commercial or financial relationships that could be construed as a potential conflict of interest.

## Publisher’s Note

All claims expressed in this article are solely those of the authors and do not necessarily represent those of their affiliated organizations, or those of the publisher, the editors and the reviewers. Any product that may be evaluated in this article, or claim that may be made by its manufacturer, is not guaranteed or endorsed by the publisher.
