# Supplementary material for: Co-expression Mechanism Analysis of Different Tachyplesin I–Resistant Strains in Pseudomonas aeruginosa Based on Transcriptome Sequencing
Source: Front Microbiol. 2022 Apr 7;13:871290. doi: 10.3389/fmicb.2022.871290 (PMC9022664; doi:10.3389/fmicb.2022.871290)
Supplement: Supplementary file 1 [file Data_Sheet_1.doc]

**Supplementary Material Caption**

**Supplementary Figure**

**Figure. S1 Box diagram of RPKM distribution of each strain.** T01-T05 stands for three biological duplications of PA1.2620 strain, T06-T08 stands for three biological duplications of tachyplesin I-resistant PA-60 strain, T09-T11 stands for three biological duplications of tachyplesin I-resistant PA-99 strain.

**Supplementary Table**

Table 1. Transcriptome sequencing summary for different *P. aeruginosa* strains.

Table 2. Statistical reads of Clean Data mapped to reference genome.

Table 3. Correlation statistics of biological duplicate.

Table 4. The enriched GO terms in HL treatments.

Table 5. The top twenty enriched pathways in HL treatments.

Table 6A. The enriched GO terms in co-expressed sRNA target genes.

Table 6B. The top twenty enriched pathways in co-expressed sRNA target genes.
