# Supplementary figures and images for: Co-expression Mechanism Analysis of Different Tachyplesin I–Resistant Strains in Pseudomonas aeruginosa Based on Transcriptome Sequencing
Source: Front Microbiol. 2022 Apr 7;13:871290. doi: 10.3389/fmicb.2022.871290 (PMC9022664; doi:10.3389/fmicb.2022.871290)

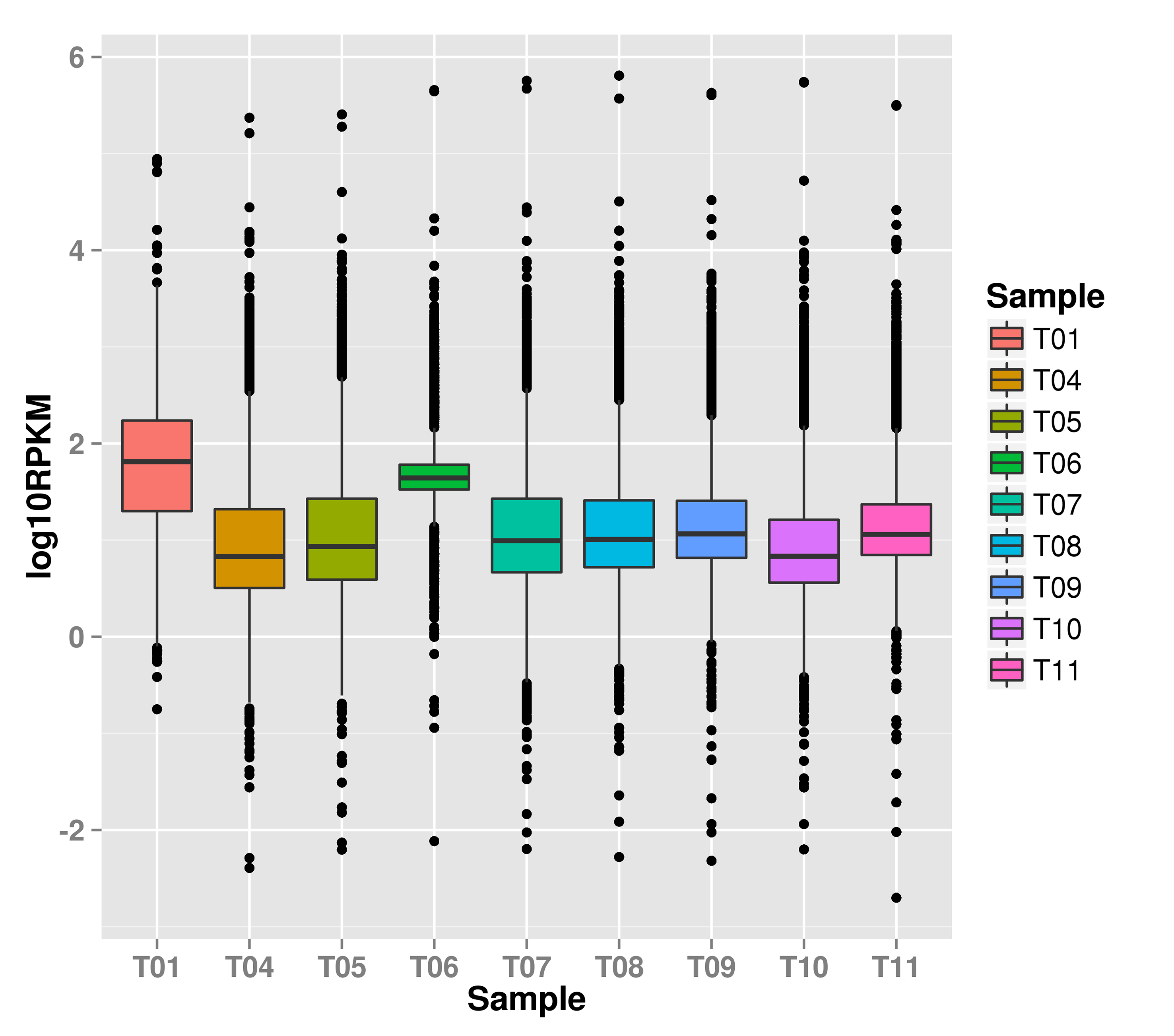

Supplement: Supplementary file 2 [file Image_1.png]
