# Supplementary material for: Co-expression Mechanism Analysis of Different Tachyplesin I–Resistant Strains in Pseudomonas aeruginosa Based on Transcriptome Sequencing
Source: Front Microbiol. 2022 Apr 7;13:871290. doi: 10.3389/fmicb.2022.871290 (PMC9022664; doi:10.3389/fmicb.2022.871290)
Supplement: Supplementary file 3 [file Table_1.docx]

**Supplementary** Table 1. Transcriptome sequencing summary for different *P. aeruginosa* strains

| **Sample ID** | **ReadSum** | **BaseSum** | **GC (%)** | **Q30 (%)** |
| --- | --- | --- | --- | --- |
| PA1.2620 ^1^ | 12,794,030 | 3,699,522,416 | 64.49 | 96.43 |
| PA1.2620^2^ | 14,442,212 | 4,311,426,684 | 63.34 | 95.46 |
| PA1.2620^3^ | 12,387,552 | 3,694,243,854 | 63.73 | 96.21 |
| PA-60^1^ | 12,687,235 | 3,784,430,848 | 64.75 | 96.27 |
| PA-60^2^ | 10,903,152 | 3,252,265,890 | 63.83 | 96.47 |
| PA-60^3^ | 7,077,582 | 2,113,068,096 | 63.50 | 96.80 |
| PA-99^1^ | 10,524,519 | 3,139,186,296 | 63.65 | 96.32 |
| PA-99^2^ | 10,602,206 | 3,161,091,324 | 62.57 | 95.60 |
| PA-99^3^ | 11,337,636 | 3,384,373,320 | 63.54 | 95.42 |

Note: Superscripted 1-3 stands for three biological duplications of one sample, the same below. Sample ID: the uniform number of the sample; ReadSum: total number of pair end Reads in Clean Data; BaseSum: clean data total base number; GC (%): Clean data GC content, that is, the percentage of G and C bases in Clean Data in total bases; Q30 (%): the percentage of bases with a clean data mass value greater than or equal to 30.
