# Supplementary material for: Co-expression Mechanism Analysis of Different Tachyplesin I–Resistant Strains in Pseudomonas aeruginosa Based on Transcriptome Sequencing
Source: Front Microbiol. 2022 Apr 7;13:871290. doi: 10.3389/fmicb.2022.871290 (PMC9022664; doi:10.3389/fmicb.2022.871290)
Supplement: Supplementary file 4 [file Table_2.docx]

**Supplementary** Table 2. Statistical reads of Clean Data mapped to reference genome

| **Sample ID** | **Total reads** | **Mapped reads** | **Unmapped reads** | **Multiple mapped reads** | **Unique mapped reads** |
| --- | --- | --- | --- | --- | --- |
| PA1.2620 ^1^ | 25588060 | 23568822  (92.11%) | 2019238 (7.89%) | 514967  (2.01%) | 23053855  (90.10%) |
| PA1.2620^2^ | 28884424 | 26610272  (92.13%) | 2274152 (7.87%) | 1596802  (5.53%) | 25013470  (86.60%) |
| PA1.2620^3^ | 24775104 | 22686017  (91.57%) | 2089087 (8.43%) | 891827  (3.60%) | 21794190  (87.97%) |
| PA-60^1^ | 25374470 | 23810467  (93.84%) | 1564003  (6.16%) | 273739  (1.08%) | 23536728  (92.76%) |
| PA-60^2^ | 21806304 | 20434536  (93.71%) | 1371768  (6.29%) | 230223  (1.06%) | 20204313  (92.65%) |
| PA-60^3^ | 14155164 | 13366929  (94.43%) | 788235  (5.57%) | 264632  (1.87%) | 13102297  (92.56%) |
| PA-99^1^ | 21049038 | 18448745  (87.65%) | 2600293 (12.35%) | 451663  (2.15%) | 17997082  (85.50%) |
| PA-99^2^ | 21204412 | 19832210  (93.53%) | 1372202 (6.47%) | 870487  (4.11%) | 18961723  (89.42%) |
| PA-99^3^ | 22675272 | 19875769  (87.65%) | 2799503 (12.35%) | 1060369  (4.68%) | 18815400  (82.98%) |
