# Supplementary material for: Co-expression Mechanism Analysis of Different Tachyplesin I–Resistant Strains in Pseudomonas aeruginosa Based on Transcriptome Sequencing
Source: Front Microbiol. 2022 Apr 7;13:871290. doi: 10.3389/fmicb.2022.871290 (PMC9022664; doi:10.3389/fmicb.2022.871290)
Supplement: Supplementary file 5 [file Table_3.docx]

**Supplementary** Table 3. Correlation statistics of biological duplicate

| **sample1** | **sample2** | **R^2^** |
| --- | --- | --- |
| PA1.2620^1^ | PA1.2620^2^ | 0.4689 |
| PA1.2620^1^ | PA1.2620^3^ | 0.4735 |
| PA1.2620^2^ | PA1.2620^3^ | 0.9931 |
| PA-60^1^ | PA-60^2^ | 0.9889 |
| PA-60^1^ | PA-60^3^ | 0.9215 |
| PA-60^2^ | PA-60^3^ | 0.968 |
| PA-99^1^ | PA-99^2^ | 0.9982 |
| PA-99^1^ | PA-99^3^ | 0.9977 |
| PA-99^2^ | PA-99^3^ | 0.997 |

Note: Deleted the biological replicates PA1.2620^1^ and PA-60^1^, respectively.
