# Supplementary material for: Co-expression Mechanism Analysis of Different Tachyplesin I–Resistant Strains in Pseudomonas aeruginosa Based on Transcriptome Sequencing
Source: Front Microbiol. 2022 Apr 7;13:871290. doi: 10.3389/fmicb.2022.871290 (PMC9022664; doi:10.3389/fmicb.2022.871290)
Supplement: Supplementary file 6 [file Table_4.doc]

**Supplementary**

Table 4. The top twenty enriched GO terms in HL treatments.

| GO_ID | GO_term | ontology | Items | Background_Items | p_adj | pvalue | Gene_ID |
| --- | --- | --- | --- | --- | --- | --- | --- |
| GO:0016652 | oxidoreductase activity, acting on NAD(P)H, NAD(P) as acceptor | Molecular Function | 3 | 3 | 0.245130128 | 0.0007882 | gene797;  gene1999;  gene3281 |
| GO:0016661 | oxidoreductase activity, acting on other nitrogenous compounds as donors | Molecular Function | 3 | 3 | 0.245130128 | 0.0007882 | gene797;  gene1999;  gene3281 |
| GO:0030983 | mismatched DNA binding | Molecular Function | 3 | 5 | 1 | 0.006834839 | Novel_828;  gene5061;  Novel_829 |
| GO:0008752 | FMN reductase activity | Molecular Function | 3 | 5 | 1 | 0.006834839 | gene3281;  gene797;  gene1999 |
| GO:0006298 | mismatch repair | Biological Process | 3 | 5 | 1 | 0.006954032 | Novel_828;  gene5061;  Novel_829 |
| GO:0004460 | L-lactate dehydrogenase (cytochrome) activity | Molecular Function | 2 | 2 | 1 | 0.008552677 | gene4883;  gene2419 |
| GO:0006880 | intracellular sequestering of iron ion | Biological Process | 2 | 2 | 1 | 0.008656902 | gene4307;  gene3598 |
| GO:0009432 | SOS response | Biological Process | 4 | 12 | 1 | 0.020043299 | gene3058;  gene3059;  Novel_164;  gene3407 |
| GO:0004488 | methylenetetrahydrofolate dehydrogenase (NADP+) activity | Molecular Function | 2 | 3 | 1 | 0.02408163 | Novel_844;  gene1827 |
| GO:0004635 | phosphoribosyl-AMP cyclohydrolase activity | Molecular Function | 2 | 3 | 1 | 0.02408163 | gene655;  Novel_861 |
| GO:0034040 | lipid-transporting ATPase activity | Molecular Function | 2 | 3 | 1 | 0.02408163 | Novel_837;  Novel_838 |
| GO:0004540 | ribonuclease activity | Molecular Function | 2 | 3 | 1 | 0.02408163 | gene3937;  Novel_187 |
| GO:0006431 | methionyl-tRNA aminoacylation | Biological Process | 2 | 3 | 1 | 0.024365372 | gene17;  Novel_525 |
| GO:0006869 | lipid transport | Biological Process | 2 | 3 | 1 | 0.024365372 | Novel_838; Novel_837 |
| GO:0000105 | histidine biosynthetic process | Biological Process | 5 | 19 | 1 | 0.026525523 | Novel_825; Novel_862;  gene1827; Novel_877;  Novel_861 |
| GO:0006413 | translational initiation | Biological Process | 3 | 8 | 1 | 0.031489398 | gene966; gene17;  Novel_795 |
| GO:0046677 | response to antibiotic | Biological Process | 7 | 34 | 1 | 0.034041455 | gene1996; gene2527; gene4649; gene472; gene4440; gene4692; gene4888 |
| GO:0009372 | quorum sensing | Biological Process | 4 | 14 | 1 | 0.034925983 | Novel_523; gene1456; Novel_522; Novel_521 |
| GO:0009423 | chorismate biosynthetic process | Biological Process | 4 | 14 | 1 | 0.034925983 | Novel_473; gene2993; gene24; Novel_472 |
| GO:0006396 | RNA processing | Biological Process | 3 | 9 | 1 | 0.044038199 | Novel_702; Novel_745; Novel_793 |
