# Supplementary material for: Co-expression Mechanism Analysis of Different Tachyplesin I–Resistant Strains in Pseudomonas aeruginosa Based on Transcriptome Sequencing
Source: Front Microbiol. 2022 Apr 7;13:871290. doi: 10.3389/fmicb.2022.871290 (PMC9022664; doi:10.3389/fmicb.2022.871290)
Supplement: Supplementary file 7 [file Table_5.doc]

**Supplementary Table 5**

**Table 5. The top twenty enriched pathways in HL treatments.**

| #Kegg_pathway | ko_id | Cluter_frequency | Genome_frequency | P-value | Corrected_P-value |
| --- | --- | --- | --- | --- | --- |
| RNA degradation | ko03018 | 8 out of 186 4.3010752688172% | 23 out of 1946 1.18191161356629% | 0.000834782 | 0.059269537 |
| Aminoacyl-tRNA biosynthesis | ko00970 | 12 out of 186 6.45161290322581% | 50 out of 1946 2.5693730729702% | 0.001903796 | 0.135169509 |
| Aminobenzoate degradation | ko00627 | 7 out of 186 3.76344086021505% | 21 out of 1946 1.07913669064748% | 0.002369675 | 0.168246929 |
| Phenylalanine, tyrosine and tryptophan biosynthesis | ko00400 | 8 out of 186 4.3010752688172% | 37 out of 1946 1.90133607399794% | 0.020418895 | 1 |
| Histidine metabolism | ko00340 | 7 out of 186 3.76344086021505% | 35 out of 1946 1.79856115107914% | 0.043453523 | 1 |
| Bacterial chemotaxis | ko02030 | 10 out of 186 5.37634408602151% | 60 out of 1946 3.08324768756423% | 0.054305088 | 1 |
| Mismatch repair | ko03430 | 5 out of 186 2.68817204301075% | 23 out of 1946 1.18191161356629% | 0.061318366 | 1 |
| Styrene degradation | ko00643 | 3 out of 186 1.61290322580645% | 13 out of 1946 0.668036998972251% | 0.120294269 | 1 |
| Glutathione metabolism | ko00480 | 6 out of 186 3.2258064516129% | 37 out of 1946 1.90133607399794% | 0.13508569 | 1 |
| Novobiocin biosynthesis | ko00401 | 2 out of 186 1.0752688172043% | 7 out of 1946 0.359712230215827% | 0.138577665 | 1 |
| Phenylalanine metabolism | ko00360 | 5 out of 186 2.68817204301075% | 30 out of 1946 1.54162384378212% | 0.151978584 | 1 |
| Riboflavin metabolism | ko00740 | 3 out of 186 1.61290322580645% | 16 out of 1946 0.822199383350462% | 0.191511762 | 1 |
| Folate biosynthesis | ko00790 | 4 out of 186 2.1505376344086% | 26 out of 1946 1.3360739979445% | 0.23246769 | 1 |
| Porphyrin and chlorophyll metabolism | ko00860 | 7 out of 186 3.76344086021505% | 54 out of 1946 2.77492291880781% | 0.253062924 | 1 |
| Phosphonate and phosphinate metabolism | ko00440 | 2 out of 186 1.0752688172043% | 11 out of 1946 0.565262076053443% | 0.283767243 | 1 |
| Homologous recombination | ko03440 | 4 out of 186 2.1505376344086% | 29 out of 1946 1.49023638232271% | 0.298421284 | 1 |
| Fructose and mannose metabolism | ko00051 | 3 out of 186 1.61290322580645% | 21 out of 1946 1.07913669064748% | 0.325005333 | 1 |
| Terpenoid backbone biosynthesis | ko00900 | 3 out of 186 1.61290322580645% | 21 out of 1946 1.07913669064748% | 0.325005333 | 1 |
| Arachidonic acid metabolism | ko00590 | 1 out of 186 0.537634408602151% | 4 out of 1946 0.205549845837616% | 0.331136285 | 1 |
| Citrate cycle (TCA cycle) | ko00020 | 6 out of 186 3.2258064516129% | 50 out of 1946 2.5693730729702% | 0.342422495 | 1 |
