# Supplementary material for: Co-expression Mechanism Analysis of Different Tachyplesin I–Resistant Strains in Pseudomonas aeruginosa Based on Transcriptome Sequencing
Source: Front Microbiol. 2022 Apr 7;13:871290. doi: 10.3389/fmicb.2022.871290 (PMC9022664; doi:10.3389/fmicb.2022.871290)
Supplement: Supplementary file 8 [file Table_6.doc]

**Supplementary Table 6**

Table 6A. The top twenty enriched GO terms in co-expressed sRNA target genes

| GO_ID | GO_term | ontology | Items | Background_Items | p_adj | pvalue | Gene_ID |
| --- | --- | --- | --- | --- | --- | --- | --- |
| GO:0016884 | carbon-nitrogen ligase activity, with glutamine as amido-N-donor | Molecular Function | 2 | 7 | 0.205764674 | 0.003117647 | gene4235; gene714 |
| GO:0006779 | porphyrin-containing compound biosynthetic process | Biological Process | 2 | 7 | 0.181159626 | 0.003123442 | gene2995; gene514 |
| GO:0008295 | spermidine biosynthetic process | Biological Process | 2 | 9 | 0.305594783 | 0.005268876 | gene4885; gene4886 |
| GO:0022904 | respiratory electron transport chain | Biological Process | 2 | 11 | 0.459430145 | 0.007921209 | gene3643; gene936 |
| GO:0004040 | amidase activity | Molecular Function | 2 | 12 | 0.621209205 | 0.009412261 | gene714; gene4235 |
| GO:0004479 | methionyl-tRNA formyltransferase activity | Molecular Function | 1 | 1 | 0.828321099 | 0.01255032 | gene17 |
| GO:0015098 | molybdate ion transmembrane transporter activity | Molecular Function | 1 | 1 | 0.828321099 | 0.01255032 | gene491 |
| GO:0004637 | phosphoribosylamine-glycine ligase activity | Molecular Function | 1 | 1 | 0.828321099 | 0.01255032 | gene4968 |
| GO:0042916 | alkylphosphonate transport | Biological Process | 1 | 1 | 0.728608675 | 0.012562219 | gene3442 |
| GO:0009454 | aerotaxis | Biological Process | 1 | 1 | 0.728608675 | 0.012562219 | gene1589 |
| GO:0019596 | mandelate catabolic process | Biological Process | 1 | 1 | 0.728608675 | 0.012562219 | gene5014 |
| GO:0071951 | conversion of methionyl-tRNA to N-formyl-methionyl-tRNA | Biological Process | 1 | 1 | 0.728608675 | 0.012562219 | gene17 |
| GO:0006089 | lactate metabolic process | Biological Process | 1 | 1 | 0.728608675 | 0.012562219 | gene4883 |
| GO:0005515 | protein binding | Molecular Function | 2 | 14 | 0.842894994 | 0.012771136 | gene1744; gene3788 |
| GO:0036054 | protein-malonyllysine demalonylase activity | Molecular Function | 1 | 2 | 1 | 0.024946064 | gene1297 |
| GO:0036055 | protein-succinyllysine desuccinylase activity | Molecular Function | 1 | 2 | 1 | 0.024946064 | gene1297 |
| GO:0031177 | phosphopantetheine binding | Molecular Function | 1 | 2 | 1 | 0.024946064 | gene2460 |
| GO:0042242 | cobyrinic acid a,c-diamide synthase activity | Molecular Function | 1 | 2 | 1 | 0.024946064 | gene1297 |
| GO:0008764 | UDP-N-acetylmuramoylalanine-D-glutamate ligase activity | Molecular Function | 1 | 2 | 1 | 0.024946064 | gene4968 |
| GO:0004460 | L-lactate dehydrogenase (cytochrome) activity | Molecular Function | 1 | 2 | 1 | 0.024946064 | gene4883 |

Table 6B. The top twenty enriched pathways in co-expressed sRNA target genes.

| #Kegg_pathway | ko_id | Cluter_frequency | Genome_frequency | P-value | Corrected_P-value |
| --- | --- | --- | --- | --- | --- |
| Aminobenzoate degradation | ko00627 | 3 out of 26 11.5384615384615% | 21 out of 1946 1.07913669064748% | 0.002402277 | 0.069666029 |
| Phenylalanine metabolism | ko00360 | 3 out of 26 11.5384615384615% | 30 out of 1946 1.54162384378212% | 0.006770213 | 0.196336173 |
| Styrene degradation | ko00643 | 2 out of 26 7.69230769230769% | 13 out of 1946 0.668036998972251% | 0.012234758 | 0.354807978 |
| Arginine and proline metabolism | ko00330 | 4 out of 26 15.3846153846154% | 86 out of 1946 4.41932168550874% | 0.025252579 | 0.732324802 |
| Tryptophan metabolism | ko00380 | 2 out of 26 7.69230769230769% | 32 out of 1946 1.64439876670092% | 0.06663892 | 1 |
| Cysteine and methionine metabolism | ko00270 | 2 out of 26 7.69230769230769% | 42 out of 1946 2.15827338129496% | 0.106718259 | 1 |
| Phosphonate and phosphinate metabolism | ko00440 | 1 out of 26 3.84615384615385% | 11 out of 1946 0.565262076053443% | 0.137864559 | 1 |
| Porphyrin and chlorophyll metabolism | ko00860 | 2 out of 26 7.69230769230769% | 54 out of 1946 2.77492291880781% | 0.161152008 | 1 |
| Bacterial chemotaxis | ko02030 | 2 out of 26 7.69230769230769% | 60 out of 1946 3.08324768756423% | 0.1900677 | 1 |
| Geraniol degradation | ko00281 | 1 out of 26 3.84615384615385% | 17 out of 1946 0.873586844809866% | 0.205160735 | 1 |
| DNA replication | ko03030 | 1 out of 26 3.84615384615385% | 18 out of 1946 0.92497430626927% | 0.215873965 | 1 |
| RNA degradation | ko03018 | 1 out of 26 3.84615384615385% | 23 out of 1946 1.18191161356629% | 0.267390665 | 1 |
| One carbon pool by folate | ko00670 | 1 out of 26 3.84615384615385% | 23 out of 1946 1.18191161356629% | 0.267390665 | 1 |
| Mismatch repair | ko03430 | 1 out of 26 3.84615384615385% | 23 out of 1946 1.18191161356629% | 0.267390665 | 1 |
| Nicotinate and nicotinamide metabolism | ko00760 | 1 out of 26 3.84615384615385% | 25 out of 1946 1.2846865364851% | 0.287072373 | 1 |
| beta-Lactam resistance | ko01501 | 1 out of 26 3.84615384615385% | 26 out of 1946 1.3360739979445% | 0.296721576 | 1 |
| Pyruvate metabolism | ko00620 | 2 out of 26 7.69230769230769% | 83 out of 1946 4.26515930113052% | 0.30523182 | 1 |
| beta-Alanine metabolism | ko00410 | 1 out of 26 3.84615384615385% | 27 out of 1946 1.38746145940391% | 0.306245138 | 1 |
| Homologous recombination | ko03440 | 1 out of 26 3.84615384615385% | 29 out of 1946 1.49023638232271% | 0.324921606 | 1 |
| Bacterial secretion system | ko03070 | 2 out of 26 7.69230769230769% | 96 out of 1946 4.93319630010278% | 0.370095452 | 1 |
